# The effects of a 4-week protein overfeeding protocol on body composition and influence of FTO genotype in exercise-trained men and women

**DOI:** 10.1080/15502783.2026.2744348

**Published:** 2026-11-04

**Authors:** Siavash Naddafha, Antonio Cristani, Kendall Andries, Kristiina Kinnunen, Lia Jiannine, Jaime Tartar, Jose Antonio, Cassandra Evans

**Affiliations:** a School of Medical and Health Sciences, Edith Cowan University, Joondalup, WA, Australia; b Department of Health and Human Performance, Nova Southeastern University, Davie, FL, USA; c Department of Neuroscience, University of Miami Miller School of Medicine, Miami, FL, USA; d Department of Psychology and Neuroscience, Nova Southeastern University, Davie, FL, USA

**Keywords:** Protein overfeeding, high-protein diet, body composition, lean body mass, fat mass, exercise-trained

## Abstract

**Background:**

High-protein diets have consistently been associated with favorable changes in body composition in both sedentary and exercise-trained populations. Despite abundant evidence supporting protein intakes above the recommended dietary allowance (RDA) for active individuals, the effects of deliberate protein overfeeding, particularly in those already consuming adequate protein, remain incompletely characterized. The primary purpose of this study was to examine the effects of a 4-week protein overfeeding protocol on body composition in exercise-trained men and women. The secondary purpose was to determine whether body composition adaptations differed by *FTO* rs9939609 genotype.

**Methods:**

Fifty-six exercise-trained men and women (age: 22.40 ± 5.78 years; body mass: 75.47 ± 14.98 kg; BMI: 24.52 ± 3.50 kg/m^2^; resistance training: 5.12 ± 3.35 h/week) were enrolled in a 4-week dietary intervention. Participants were instructed to increase their daily protein intake by approximately 20% above baseline while maintaining habitual training. Body composition was assessed pre- and post-intervention using a multi-frequency bioelectrical impedance device (InBody® 270). Dietary intake was self-monitored via MyFitnessPal®. Thirty-five participants completed both pre- and post-testing assessments and constituted the primary analysis. Linear regression models adjusted for baseline values indicated that FTO genotype was not a significant predictor of body composition changes (*p* > 0.05).

**Results:**

Protein intake trended toward a significant increase over the 4-week period (pre: 146 ± 66 g/day vs. post: 164 ± 79 g/day; *p* = 0.053), representing an approximate 12% increase. Total energy intake, carbohydrate, and fat intake did not significantly change. Lean body mass increased significantly (pre: 59.95 ± 14.14 kg vs. post: 60.62 ± 14.42 kg; *p* = 0.005; Cohen's d = 0.31) and total body water increased significantly (pre: 43.85 ± 10.35 L vs. post: 44.34 ± 10.55 L; *p* = 0.005). Fat mass (*p* = 0.424), body fat percentage (*p* = 0.149), and total body mass (*p* = 0.09) did not significantly change.

**Conclusions:**

A 4-week modest protein overfeeding protocol in exercise-trained men and women was associated with a significant increase in lean body mass and total body water without concomitant increases in fat mass. These adaptations were not modified by FTO rs9939609 genotype. These findings support the contention that higher protein intakes do not promote fat accretion and may facilitate improvements in body composition in trained individuals.

## 1. Background

Body composition is a key indicator of overall health and provides a more comprehensive assessment of nutritional status than body mass index (BMI) alone. Excess body fat is associated with an increased risk of noncommunicable diseases, including cardiovascular disease, type 2 diabetes, and metabolic syndrome, whereas greater lean body mass is associated with improved health outcomes and lower mortality risk [1–4]. Changes in body composition are influenced by numerous factors, including dietary intake, physical activity, and genetics. Diets characterized by high intake of energy-dense, ultra-processed foods, saturated fat, and added sugars are associated with increased body fat and a greater risk of obesity and chronic disease. Conversely, nutrient and protein-dense dietary patterns may promote healthier body composition [1,2] and correlate with higher LBM [5–7]. Notably, substantial interindividual variability exists in responses to dietary interventions affecting body composition, suggesting that genetic variation may contribute to differences in obesity risk and metabolic outcomes.

Genome-wide association studies (GWAS) have identified more than 300 single nucleotide- polymorphisms (SNPs) associated with obesity. Among these, variants within the FTO alpha-ketoglutarate-dependent dioxygenase (FTO) gene demonstrate the strongest and most consistently replicated association with obesity across diverse populations [8,9]. The most extensively studied obesity-associated variants are located within the first intron (non-coding gene segment removed before translation) of the FTO gene, including rs9939609 (A/T), rs17817449 (G/T), rs3751812 (T/G), and rs1421085 (C/T) [8–12]. Individuals with the risk variants on this gene are more likely to suffer from obesity [8–12]. Several studies have reported that individuals carrying FTO risk alleles consume greater amounts of energy, particularly from dietary fat and sugar, and exhibit reduced satiety compared with non-carriers [13–25]. For example, Harbron et al [15] investigated the association between FTO variants (C-, G-, and T-alleles) and dietary intake, eating behaviors, physical activity, and psychological health. They reported that individuals carrying the GG risk allele consumed significantly greater amounts of saturated fat and refined sugars than those without the risk allele [15]. While research suggests carrying the FTO SNP does not inhibit or stunt an individual’s ability to lose weight when following a hypocaloric diet [26–28], it is uncertain if FTO genotype influences the response to dietary interventions involving overfeeding.

Protein is an essential macronutrient critical for muscle protein synthesis, repair, and maintenance of lean body mass. The current recommended dietary allowance (RDA) for protein is 0.8 g/kg/day; however, mounting evidence indicates that this level is insufficient for individuals who engage in regular exercise [29,30]. Position statements from the Academy of Nutrition and Dietetics, Dietitians of Canada, and the American College of Sports Medicine recommend protein intakes of 1.2–2.0 g/kg/day for active individuals [29]. Even higher intakes have been investigated and well-tolerated in resistance-trained populations [5,6]. Several investigations by Antonio and colleagues have examined the effects of consuming very high-protein diets, ranging from 2.6 to 4.4 g/kg/day, in resistance-trained individuals [5,6]. These studies consistently demonstrated that substantial protein overfeeding did not result in significant increases in fat mass, even when total energy intake was elevated. A plausible mechanistic explanation is the thermic effect of protein. Diet-induced thermogenesis is proportionally greatest for protein (20–30%) compared to carbohydrate (5–10%) or fat (0–3%), such that increases in dietary protein may substantially elevate total daily energy expenditure, partially or fully offsetting the caloric surplus [31,32]. Oliveira et al. demonstrated that a high-protein total diet replacement increased energy expenditure and produced a negative fat balance in healthy, normal-weight adults [31], further supporting the thermogenic properties of dietary protein. Despite this body of work, relatively few studies have examined the effects of modest, rather than extreme, protein overfeeding on body composition in already-trained individuals who consume habitual protein intakes near or above current recommendations. Whether a moderate increase in protein intake above an already-adequate baseline is sufficient to produce favorable shifts in body composition remains an open question. Furthermore, prior research has been largely characterized by cross-sectional or self-selected dietary patterns, making it difficult to isolate the specific contribution of dietary protein to changes in body composition.

Therefore, the primary purpose of the present study was to examine the effects of a 4-week protein overfeeding intervention, in which exercise-trained men and women were instructed to increase protein intake by approximately 20% above their habitual intake, on body composition outcomes. The secondary outcome was to determine whether changes in body composition differed between individuals carrying the FTO risk genotype and those with the non-risk genotype. We hypothesized that a moderate increase in dietary protein would increase lean body mass without promoting fat accumulation and that the response to protein overfeeding may differ according to FTO genotype.

## 2. Methods

### 2.1. Participants

A convenience sample of healthy, exercise-trained adults (minimum of one year of regular physical activity) was recruited from a university community. Regular physical activity was defined as a minimum of 150 minutes of moderate aerobic activity per week or muscle-strengthening exercises on at least two days per week [33]. Exclusion criteria included any current use of anabolic steroids, current injury precluding normal training, or a self-reported history of kidney or liver disease. In accordance with the Declaration of Helsinki, the institutional review board approved all study procedures involving human participants. Written informed consent was obtained from all participants prior to enrollment. Fifty-six participants were enrolled and completed baseline testing; of these, 35 completed post-intervention assessments and constituted the primary analysis.

### 2.2. Experimental design

This investigation employed a single-group, pre-post design. Participants attended the laboratory on two occasions: once at baseline (pre) and once following a 4-week dietary intervention (post). At each visit, body composition was assessed, dietary intake was reviewed, and anthropometric measurements were recorded. All testing took place between 11:30 and 14:00 to control for circadian variation. Participants were instructed to avoid exercise, food, and caloric beverages for at least three hours prior to each testing session.

During the 4-week treatment period, participants were instructed to increase their daily protein intake by approximately 20% above their individual baseline intake while maintaining their habitual training regimen. Participants were offered a commercially available protein powder (MRM Whey Protein, MRM Nutrition, Oceanside, CA, USA; MuscleTech ISO Whey Protein Isolate, MuscleTech, Boca Raton, FL, USA; and ProTYM Whey Isolate, TYM Athletic Performance, Dallas, TX, USA) to facilitate adherence; however, they were free to achieve the prescribed increase using whole foods, protein supplements, or any combination of these sources. Participants were specifically asked not to meaningfully alter their carbohydrate or fat intake so that the primary dietary manipulation remained focused on protein.

### 2.3. Dietary assessment

Participants self-monitored their dietary intake using the MyFitnessPal® smartphone application (Under Armor, Inc., Baltimore, MD, USA) for a minimum of three days per week throughout the study. The validity of mobile application-based dietary tracking has been previously established [34]. The MyFitnessPal® database contains over five million foods sourced from user input, barcode scanning, and the USDA National Nutrient Database. Baseline dietary data were derived from pre-existing food logs to characterize habitual intake. Average daily energy, protein, carbohydrate, and fat intakes were calculated for the pre- and post-intervention periods.

### 2.4. Body composition assessment

Body composition was assessed at each visit using a validated, multi-frequency bioelectrical impedance analysis (BIA) device (InBody® 270; InBody USA, Cerritos, CA). Participants stood on the platform of the device barefoot with the soles of their feet in contact with the foot electrodes. They then grasped the hand electrodes with their thumbs and fingers, maintaining direct electrode contact with both hands. Participants stood still for approximately one minute with elbows fully extended and shoulder joints abducted to approximately 30 degrees. The device measured body mass (kg), body mass index (BMI; kg/m^2^), fat mass (kg), lean body mass (LBM; kg), body fat percentage (%), and total body water (TBW; L). Segmental body circumferences (waist, mid-arm, and mid-thigh) were also assessed. Mid-thigh circumference was measured on the right side at the midpoint between the inguinal crease and the proximal border of the patella. Waist circumference was measured at the narrowest point between the ribs and hips following a normal exhalation. Mid-arm circumference was measured at the midpoint between the acromion and elbow crease with the arm relaxed.

### 2.5. Genotyping

Genomic DNA was extracted using a QIAcube instrument following the manufacturer’s standard protocol for saliva nucleic acid extraction (QIAGEN, Valencia, CA). After isolation, allelic discrimination for the FTO SNPs was determined via real-time polymerase chain reaction (PCR) using TaqMan SNP genotyping assays using fluorogenic probes (Applied Biosystems, CA) with the following primer sequences.

rs9939609:

GGTTCCTTGCGACTGCTGTGAATTT [A/T] GTGATGCACTTGGATAGTCTCTGTT.

Thermal cycling was performed on the StepOne Real-Time PCR system (Applied Biosystems, CA). The amplification mix contained the following ingredients: 12.5 μL of PCR master mix (QIAGEN, Valencia, CA), 1.25 μL of TaqMan 20X working stock, 10.25 μL of RNase- and DNase-free water (Sigma), and 1.0 μL of sample DNA, in a total volume of 25 μL per single tube reaction. The PCR condition was 95 °C for 10 min followed by 40 repeated cycles of 95 °C for 15 s and 60 °C for 60 s. Genotypes were determined automatically via the StepOne software (Applied Biosystems, CA) based on the fluorescence signals. Samples were run in duplicate, and in the case of a call discrepancy, samples were rerun. Participants with the normal obesity risk are classified as “non-risk allele” versus those that carry the risk as “risk allele”. We have classified those with the normal obesity risk (T/T) as “non-risk allele” versus those that carry the “risk allele” (A/T and A/A) (i.e. both heterozygous and homozygous).

### 2.6. Statistical analysis

All data are presented as mean ± standard deviation (SD). Paired-samples t-tests were used to assess pre-to-post changes in dietary intake and body composition outcomes within the completer cohort (*n* = 35). Effect sizes were calculated using Cohen's d (d = mean difference/pooled SD). All reported *p*-values are two-tailed, with an a priori significance level set at *p* < 0.05. A trend toward significance was noted when 0.05 ≤ *p* < 0.10. Statistical analyzes were performed using GraphPad Prism (version 9; GraphPad Software, San Diego, CA, USA).

## 3. Results

### 3.1. Participants

Fifty-six exercise-trained men and women completed baseline assessments. Of these, 35 participants returned for post-intervention testing and were included in the primary analysis. The 21 participants who did not complete post-testing were unavailable for follow-up or could not be contacted and were therefore excluded. Baseline characteristics of all enrolled participants (*n* = 56) are presented in Table 1. On average, participants had 7.40 ± 6.66 years of training experience, performed approximately 3.48 ± 3.59 hours of cardiovascular exercise and 5.12 ± 3.35 hours of resistance training per week, and were classified as normal weight (BMI: 24.52 ± 3.50 kg/m^2^). Based on the FTO rs9939609 genotype, 33 participants (58.9%) were classified as carriers of the risk allele (AA/AT), while 23 participants (41.1%) were classified as non-risk carriers (TT).

**Table 1.** Baseline participant characteristics (*n* = 56).

Characteristic	Mean ± SD
Age (years)	22.4 ± 5.8
Training experience (years)	7.4 ± 6.7
Cardiovascular exercise (h/week)	3.4 ± 3.5
Resistance training (h/week)	5.1 ± 3.3
Body mass index (kg/m^2^)	24.5 ± 3.5
Body mass (kg)	75.4 ± 14.9
Fat mass (kg)	14.1 ± 6.6
Lean body mass (kg)	61.0 ± 14.5
Body fat (%)	18.8 ± 8.2
Total body water (L)	44.6 ± 11.2
FTO Non-Risk Genotype, *n* (%)	23 (41.1)
FTO Risk Genotype, *n* (%)	33 (58.9)

Data are expressed as Mean ± SD.

### 3.2. Baseline FTO genotype comparisons

Baseline body composition and dietary intake variables did not differ significantly between participants with the FTO risk genotype (AA/AT) and those with the non-risk genotype (TT) (all *p* > 0.05; Table 2). Specifically, there were no significant differences in BMI (*p* = 0.867), body mass (*p* = 0.819), fat mass (*p* = 0.331), lean body mass (*p* = 0.472), body fat percentage (*p* = 0.423), or total body water (*p* = 0.869) between genotype groups. Similarly, no significant baseline differences were observed in energy intake (*p* = 0.280), carbohydrate intake (*p* = 0.189), fat intake (*p* = 0.904), or protein intake (*p* = 0.554).

**Table 2.** Baseline differences in body composition and dietary intake outcomes between FTO risk and non-risk genotype groups.

Outcome	Non-Risk Genotype (*n* = 23) Mean ± SD	Risk Genotype (*n* = 33) Mean ± SD	t-value	df	*p*-value
BMI (kg/m^2^)	24.40 ± 2.87	24.27 ± 3.05	0.168	49.242	0.867
Body Mass (kg)	75.43 ± 13.13	74.60 ± 13.40	0.230	48.107	0.819
Fat Mass (kg)	12.50 ± 5.23	13.95 ± 5.77	−0.982	50.276	0.331
Lean Body Mass (kg)	62.93 ± 13.81	60.13 ± 14.84	0.725	49.592	0.472
Body Fat (%)	17.00 ± 7.49	18.78 ± 8.72	−0.808	49.544	0.423
Total Body Water (L)	45.07 ± 12.07	44.55 ± 10.78	0.166	43.920	0.869
Energy Intake (kcal/day)	2283.68 ± 876.25	2014.79 ± 623.31	1.099	31.946	0.280
Carbohydrate Intake (g/day)	219.26 ± 106.83	184.53 ± 75.03	1.340	37.059	0.189
Fat Intake (g/day)	115.21 ± 121.86	121.27 ± 243.09	−0.122	48.169	0.904
Protein Intake (g/day)	152.99 ± 65.94	142.21 ± 66.51	0.596	47.801	0.554

### 3.3. Dietary intake

Dietary intake data (irrespective of genotype) pre- and post-intervention are presented in Table 3. Protein intake trended toward a significant increase from pre- to post-intervention (146 ± 66 g/day vs. 164 ± 79 g/day; Δ = +18 g/day; *p* = 0.053), representing an approximate 12% increase. When expressed relative to body mass, protein intake similarly trended toward an increase (1.93 ± 0.68 g/kg/day vs. 2.12 ± 0.75 g/kg/day; *p* = 0.053). Total energy intake did not significantly change (2124 ± 746 kcal/day vs. 2175 ± 826 kcal/day; Δ = +51 kcal/day; *p* = 0.691). Neither carbohydrate (*p* = 0.929) nor fat intake (*p* = 0.676) changed significantly over the 4-week period. On average, participants achieved approximately 60% of the targeted 20% protein increase.

**Table 3.** Pre- and post-intervention dietary intake (*n* = 35).

Variable	Pre	Post	Δ
Energy (kcal/day)	2124 ± 74	2175 ± 82	+51
Carbohydrate (g/day)	196 ± 9	195 ± 7	−1
Fat (g/day)	86 ± 3	89 ± 4	+3
Protein (g/day)	146 ± 6	164 ± 7	+18^†^
Protein (g/kg/day)	1.93 ± 0.6	2.12 ± 0.7	+0.19^†^

Data are expressed as Mean ± SD. Δ = post minus pre.

^†^ Trend toward significance (p = 0.053).

### 3.4. Body composition

Body composition outcomes are presented in Table 4. Lean body mass increased significantly over the 4-week period (59.95 ± 14.14 kg vs. 60.62 ± 14.42 kg; Δ = +0.67 kg; *p* = 0.005; Cohen's d = 0.31). Total body water also increased significantly (43.85 ± 10.35 L vs. 44.34 ± 10.55 L; Δ = +0.49 L; *p* = 0.005). BMI increased modestly but significantly (24.47 ± 3.91 kg/m^2^ vs. 24.66 ± 3.86 kg/m^2^; Δ = +0.19 kg/m^2^; *p* = 0.008). Total body mass trended toward an increase but did not reach statistical significance (74.46 ± 15.96 kg vs. 75.03 ± 16.20 kg; Δ = +0.57 kg; *p* = 0.09; d = 0.28). Fat mass did not significantly change (14.12 ± 7.69 kg vs. 14.49 ± 7.82 kg; *p* = 0.424; d = 0.00). Body fat percentage trended toward a decrease but was not statistically significant (19.23 ± 8.38% vs. 18.77 ± 8.45%; *p* = 0.149; d = −0.06).

**Table 4.** Pre- and post-intervention body composition (*n* = 35).

Variable	Pre	Post	Δ
Body mass (kg)	74.46 ± 15.9	75.03 ± 16.2	+0.57
BMI (kg/m^2^)	24.47 ± 3.9	24.66 ± 3.8	+0.19*
Fat mass (kg)	14.12 ± 7.6	14.49 ± 7.8	+0.37
Lean body mass (kg)	59.95 ± 14.1	60.62 ± 14.4	+0.67*
Body fat (%)	19.23 ± 8.3	18.77 ± 8.4	−0.46
Total body water (L)	43.85 ± 10.3	44.34 ± 10.5	+0.49*

Data are expressed as Mean ± SD. Δ = post minus pre.

^*^ *p* < 0.05, post vs. pre.

**Figure:**
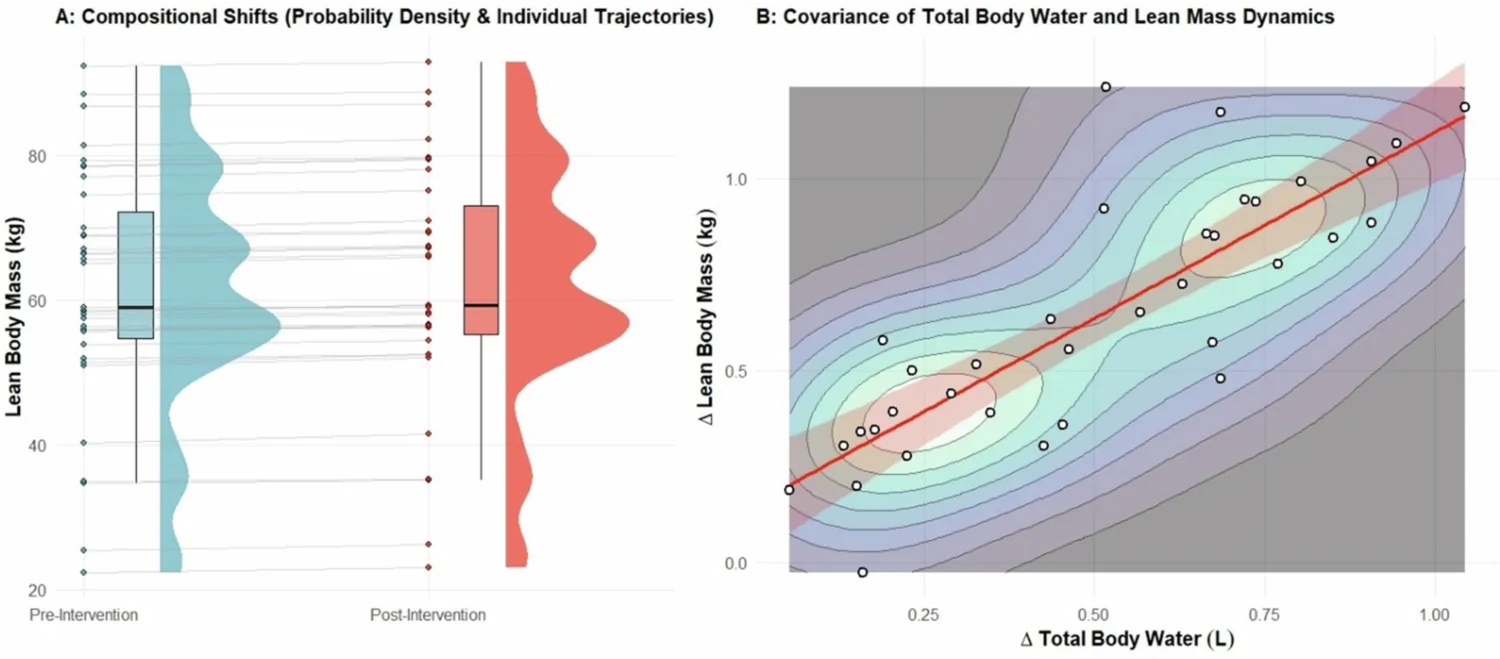


### 3.5. FTO genotype and intervention response

Linear regression models adjusted for baseline values indicated that FTO genotype was not a significant predictor of changes in body composition following the 4-week intervention (Table 5). No significant associations were observed for BMI (*β* = −0.253, *p* = 0.113), body mass (*β* = −5.593, *p* = 0.201), fat mass (*β* = 0.004, *p* = 0.993), lean body mass (*β* = −0.491, *p* = 0.362), body fat percentage (*β* = 0.751, *p* = 0.342), or total body water (*β* = −0.384, *p* = 0.323).

**Table 5.** Linear regression models for body composition adaptations predicted by *FTO* genotype (adjusted for baseline values, n=35 ).

Dependent variable (Δ)	Standardized β	*p*-value
BMI ( ${\text{kg}/\text{m}}^{2}$ )	−0.253	0.113
Body Mass (kg)	−5.593	0.201
Fat Mass (kg)	0.004	0.993
Lean Body Mass (kg)	−0.491	0.362
Body Fat (%)	0.751	0.342
Total Body Water (L)	−0.384	0.323

## 4. Discussion

The primary finding of this investigation is that a 4-week protocol designed to increase protein intake by ~20% above baseline in exercise-trained men and women resulted in a significant increase in lean body mass and total body water without a concomitant increase in fat mass. Although adherence to the targeted protein increment was partial (~12% increase achieved vs. 20% intended), even this modest elevation in dietary protein was associated with a meaningful increase in lean mass (Δ = +0.67 kg; d = 0.31). These findings are consistent with the broader body of literature suggesting that higher protein intakes facilitate favorable body composition outcomes in resistance-trained populations. The secondary finding was that usual dietary intake (assessed via baseline intake) did not differ between those with the FTO risk variant.

The Antonio laboratory has published extensively on the effects of very high-protein diets, those exceeding 3.0–4.4 g/kg/day and consistently demonstrated that extreme protein overfeeding does not lead to fat gain in resistance-trained individuals [5,6]. Our findings extend this work to a more ecologically valid context: a moderate, deliberate increase in protein intake above an alternativelyhigh habitual level. The present cohort consumed approximately 1.93 g/kg/day at baseline, already exceeding standard recommendations for active adults, and increased this to 2.12 g/kg/day. That a small increment at this range still produced a significant increase in lean mass suggests that the anabolic and thermogenic properties of dietary protein may operate even when baseline intakes are relatively high.

The significant increase in total body water (+0.49 L) likely explains, at least in part, the concurrent increase in lean body mass, as hydration status and intracellular water are primary contributors to the lean mass compartment as measured by BIA. An elevation in TBW accompanying increased protein intake is biologically plausible: Skeletal muscle is composed primarily of water, and increases in lean tissue are accompanied by corresponding increases in intracellular water [35], which likely contributed to the observed increase in total body water. This interpretation is consistent with the observation that body fat percentage did not significantly change (Δ = −0.46%; *p* = 0.149), suggesting that the lean mass gains were genuine compositional shifts rather than artifactual increases driven by edema. Furthermore, the increased body water was likely a component of skeletal muscle gain rather than mere increases in glycogen-associated intracellular water, since carbohydrate intake remained stable throughout the intervention.

Importantly, fat mass remained essentially unchanged (Δ = +0.37 kg; *p* = 0.424; d = 0.00). This null finding for fat mass is a consistent theme in the protein overfeeding literature, and the thermic effect of protein provides a compelling mechanistic basis. Protein elicits a substantially greater diet-induced thermogenesis (20–30%) compared to either carbohydrate or fat [32,36]. Accordingly, the modest increase in total energy intake observed in the present study (~51 kcal/day, *p* = 0.691) was likely offset or surpassed by the thermogenic cost of the additional protein. Oliveira et al. demonstrated in a controlled setting that a high-protein diet replacement increased total energy expenditure and produced a negative fat balance [31], and the current data corroborate these findings in a free-living exercise-trained sample.

In the present study, FTO genotype was not associated with baseline differences in body composition or dietary intake, nor did it predict changes in body composition following the 4-week protein overfeeding intervention. Although the FTO risk allele has consistently been associated with greater adiposity and obesity risk in the general population [37–39], previous studies suggest that these associations may be attenuated in physically active individuals. Several studies [40–42] report no differences in anthropometric outcomes between FTO genotype groups among physically active individuals, while a large meta-analysis estimated that regular physical activity reduces the obesity-promoting effect of the FTO risk allele by approximately 30% [43]. Collectively, these findings suggest that habitual physical activity may attenuate the FTO-associated obesity risk. The lack of genotype-dependent differences observed in the present study may therefore reflect the highly active nature of the cohort. However, because only 35 participants completed the intervention and genotype subgroup sizes were small, the study was likely underpowered to detect subtle genotype-dependent responses. Larger, adequately powered intervention studies are needed to determine whether FTO genotype modifies body composition adaptations to increased dietary protein in exercise-trained individuals.

A notable limitation of this investigation was dietary adherence. Although participants were instructed to increase protein intake by 20%, the observed increase was approximately 12%, suggesting only partial adherence to the intervention. Future studies should consider providing protein supplements or using controlled feeding protocols to improve adherence. Although investigation the effect of a change in a single variable such as protein intake may be useful, especially while participants maintained habitual training, future trials on this topic should incorporate structured, supervised, and progressive resistance training program to better explore potential adaptions to additional protein. Body composition was assessed using bioelectrical impedance analysis (BIA), which, although practical and non-invasive, is more susceptible to measurement error than criterion methods such as dual-energy X-ray absorptiometry (DXA), particularly due to fluctuations in hydration status. Future studies should incorporate DXA to improve the precision of body composition assessment. The 4-week intervention may also have been insufficient to detect larger changes in body composition, particularly in exercise-trained individuals who already possess an established anabolic stimulus. Longer interventions (12–24 weeks) may better capture meaningful changes in lean and fat mass. The relatively modest sample size of participants who completed the intervention (*n* = 35) reduced statistical power and limited the generalizability of the findings. Furthermore, generalizing these findings to other populations; such as (women, the elderly, and various disease states) mut be done with caution. This issue was further compounded in the FTO genotype analyzes, where subgroup comparisons between risk and non-risk allele carriers further reduced the effective sample size. As a result, the study may have been underpowered to detect genotype-dependent differences in body composition adaptations to protein overfeeding. Future studies should enroll larger cohorts with prospective sample size calculations and implement strategies to improve participant retention.

The present findings, interpreted alongside the existing protein overfeeding literature, suggest that a modest increase in dietary protein does not promote fat accumulation and may support increases in lean body mass in exercise-trained individuals. No significant differences in body composition responses were observed between FTO risk and non-risk genotype groups; however, the modest sample size may have limited the ability to detect genotype-dependent effects. From a practical standpoint, these findings provide reassurance to resistance-trained athletes and physically active individuals who already consume protein at or above current recommendations that modest increases in protein intake are unlikely to result in fat gain and may support lean mass accretion. Future studies with larger cohorts are needed to determine whether FTO genotype influences adaptations to protein overfeeding.

## 5. Conclusions

A 4-week protein overfeeding protocol in exercise-trained men and women resulted in a significant increase in lean body mass (+0.67 kg; *p* = 0.005) and total body water (+0.49 L; *p* = 0.005) without a significant change in fat mass or body fat percentage. Although participants achieved approximately 60% of the targeted 20% protein increase (~12% actual increase), even this modest elevation in dietary protein was associated with a small but statistically significant increase in lean body mass (Cohen's d = 0.31). These findings corroborate prior evidence that higher dietary protein intakes do not promote fat accretion and may confer favorable body composition changes, including lean mass gains, in exercise-trained populations.

## Data Availability

The datasets are available upon reasonable request.
